# Prevalence and mortality of cryptococcal disease in adults with advanced HIV in an urban tertiary hospital in Sierra Leone: a prospective study

**DOI:** 10.1186/s12879-020-4862-x

**Published:** 2020-02-14

**Authors:** Sulaiman Lakoh, Hannah Rickman, Momodu Sesay, Sartie Kenneh, Rachael Burke, Mamadu Baldeh, Darlinda F. Jiba, Yusuf S. Tejan, Sonia Boyle, Comfort Koroma, Gibrilla F. Deen, Fenella Beynon

**Affiliations:** 1 Department of Internal Medicine, College of Medicine and Allied Health Sciences, University of Sierra Leone, Freetown, Sierra Leone; 2 Ministry of Health and Sanitation, Government of Sierra Leone, Freetown, Sierra Leone; 3 King’s Sierra Leone Partnership, Freetown, Sierra Leone; 4 King’s Centre for Global Health and Health Partnerships, London, UK; 5 King’s College London, London, UK; 6 National HIV/AIDS Secretariat, Government of Sierra Leone, Freetown, Sierra Leone

**Keywords:** HIV/Cryptococcal antigenaemia/ART/newly diagnosed

## Abstract

**Background:**

The global annual estimate for cryptococcal disease-related deaths exceeds 180,000, with three fourth occurring in sub-Saharan Africa. The World Health Organization (WHO) recommends cryptococcal antigen (CrAg) screening in all HIV patients with CD4 count < 100/μl. As there is no previous published study on the burden and impact of cryptococcal disease in Sierra Leone, research is needed to inform public health policies. We aimed to establish the seroprevalence and mortality of cryptococcal disease in adults with advanced HIV attending an urban tertiary hospital in Sierra Leone.

**Methods:**

A prospective cohort study design was used to screen consecutive adult (18 years or older) HIV patients at Connaught Hospital in Freetown, Sierra Leone with CD4 count below 100 cells/mm^3^ from January to April 2018. Participants received a blood CrAg lateral flow assay (IMMY, Oklahoma, USA). All participants with a positive serum CrAg had lumbar puncture and cerebrospinal fluid (CSF) CrAg assay, and those with cryptococcal diseases had fluconazole monotherapy with 8 weeks followed up. Data were entered into Excel and analysed in Stata version 13.0. Proportions, median and interquartile ranges were used to summarise the data. Fisher’s exact test was used to compare categorical variables.

**Results:**

A total of 170 patients, with median age of 36 (IQR 30–43) and median CD4 count of 45 cells/mm^3^ (IQR 23–63) were screened. At the time of enrolment, 54% were inpatients, 51% were newly diagnosed with HIV, and 56% were either ART-naïve or newly initiated (≤ 30 days). Eight participants had a positive blood CrAg, giving a prevalence of 4.7% (95% CI: 2.4–9.2%). Of those with a positive CrAg, CSF CrAg was positive in five (62.5%). Five (62.5%) CrAg-positive participants died within the first month, while the remaining three were alive and established on ART at 8 weeks.

**Conclusion:**

A substantial prevalence of cryptococcal antigenaemia and poor outcome of cryptococcal disease were demonstrated in our study. The high mortality suggests a need for the HIV programme to formulate and implement policies on screening and pre-emptive fluconazole therapy for all adults with advanced HIV in Sierra Leone, and advocate for affordable access to effective antifungal therapies.

## Background

Disseminated cryptococcal disease, most commonly manifesting as cryptococcal meningitis, is a serious opportunistic infection in people with advanced HIV infection. Globally it is estimated to kill over 180,000 people per year, with 75% of deaths occurring in sub-Saharan Africa [1]. Mortality of cryptococcal meningitis in low-income countries may exceed 70% [1].

In addition to scaling up HIV testing and antiretroviral therapy (ART) coverage, 2018 WHO guidelines recommend two strategies for the prevention of cryptococcal meningitis [2]. The preferred option is screening patients with CD4 below 100 for cryptococcal antigen (CrAg), which has been shown to be effective and cost-effective in a variety of low-resource settings [3]^,^ [4]. However, an alternative strategy of fluconazole primary prophylaxis for patients with CD4 below 100 may also be effective in reducing cryptococcal meningitis mortality [5, 6] and is recommended where CrAg screening is not available [2].

Sierra Leone has an estimated HIV prevalence of 1.5% [7]. Despite recent ART scale-up there continues to be a significant burden of undiagnosed infection, treatment failure, and advanced disease. There is a significant human resource for health gap [8] and the majority of HIV care is nurse-led. The only available treatment for cryptococcal meningitis is fluconazole monotherapy, which is known to be associated with inferior outcomes compared to highly recommended antifungal regimens [9].

There is no previous published study of the prevalence of cryptococcal disease and related mortality in Sierra Leone, or the prevalence of cryptococcal antigen in people living with HIV (PLHIV). We, therefore, performed a cross-sectional study to analyse a pilot programme of cryptococcal antigen screening in inpatients and outpatients PLHIVs with CD4 cell count< 100/μl at an urban referral hospital in Sierra Leone. We aimed to establish the prevalence of cryptococcal antigenaemia and diseases and to assess cryptococcal disease-related mortality in people living with HIV in this setting.

## Methods

### Study setting and design

This was a prospective cohort study based in Sierra Leone’s tertiary adult referral hospital in Freetown. This acts as the central hub for HIV care in Sierra Leone, as well as a referral site for other patients nationwide. The HIV clinic serves approximately 4000 patients.

### Participant selection

Participants were recruited sequentially over 16 weeks from January to April 2018. Participants were recruited from the CD4 laboratory based in the HIV clinic, with all patients aged 18 years or older with a CD4 less than 100 cells/mm^3^ eligible for inclusion. This included inpatients and outpatients, and both newly diagnosed patients and those having CD4 cell count measured for disease monitoring or investigation of suspected opportunistic infections. Exclusion criteria were those under 18, patients with confirmed previous cryptococcal disease, and those who declined consent.

### Screening procedure

All participants with positive cryptococcal antigen were reviewed by a doctor, had a lumbar puncture unless contraindicated, and followed up for survival for a minimum of 8 weeks. Participants with asymptomatic cryptococcal antigenemia were treated with oral fluconazole monotherapy @ a dose of 800 mg daily for 2 weeks followed by 400 mg daily for 8 weeks and then 200 mg daily long-term (for at least 1 year, until CD4 > 200.) ART initiation was deferred to 2 weeks. Participants with cryptococcal meningitis were treated with fluconazole monotherapy according to national policy; oral/intravenous fluconazole monotherapy @ 1200 mg daily for 2 weeks, followed by an oral dose of 800 mg daily for 8 weeks and then continued on 200 mg daily for at least 1 year until CD4 count> 200/mm^3^. ART initiation was deferred at 4–6 weeks.

At the time of study initiation, this regimen was listed as an acceptable, though not a preferred regimen in the WHO guidelines, and is the only treatment available for cryptococcal meningitis in Sierra Leone.

### Clinical procedure

Demographic and clinical information was collected from participants using a standardised data collection form by a trained research nurse and cross-checked with clinical records. CrAg-positive patients were followed up by medical and nursing members of the research team, but responsibility for patient management was with the admitting medical consultant (inpatients) and HIV clinic staff (outpatients).

Vital status was confirmed by review or discussion with the participant or a relative by telephone (where consent was given). A post-mortem was not available, and it was therefore not possible to definitively confirm the cause of death; this was based on the clinical impression of the medical team.

### Laboratory procedures

CD4 was measured using a point-of-care PIMA analyser using whole blood from fingerpick or EDTA samples. Whole blood CrAg was measured using a commercially available Lateral Flow Assay (IMMY, Oklahoma, USA); quantitative measurement was not performed.

As a minimum, all patients had a CSF CrAg test, and measurement of opening pressure using sterile intravenous giving sets, or approximation using CSF drip rates (manometers not available) [10]. Other CSF analysis depended on the availability of tests; where formal laboratory testing was not available, some CSF parameters were approximated using urine dipstick testing of CSF [11].

### Sample size

The study was intended to follow the duration of a 16-week screening pilot. If the prevalence of cryptococcal antigen is 10% (similar to studies in Nigeria, the nearest geographical data), the sample size for 95% confidence and 5% accuracy was 138 patients. In 16 weeks, it was estimated that 160 patients would attend with CD4 < 100.

### Statistical analysis

Data were entered into Excel and analysed in Stata version 13.0(College Station, Texas, USA: StataCorp LP). Data were summarised using proportions (categorical data) and median and interquartile ranges (non-parametric continuous data). Fisher’s exact test was used to compare categorical variables.

### Ethical issues

This was an observational study in the context of the implementation of a WHO-recommended intervention (cryptococcal antigen screening). When patients had the capacity, informed written consent was sought for completion of a questionnaire and examination, and the use of anonymised clinical and demographic data. For all patients, the consent form was explained verbally in their local languages. Where patients were illiterate, the study and the consent form content were explained to them verbally and they indicated consent through a witnessed fingerprint. Patients who declined consent were still eligible for CrAg testing, but their data was not included in the analysis.

Some eligible participants lacked capacity as a result of suspected neurological opportunistic infections. The inclusion of this group was important in understanding the prevalence of cryptococcal infection in the study population. It was not felt ethical to seek consent from a representative as this would have involved the disclosure of HIV status; less than a third of people living with HIV in Sierra Leone have disclosed their status to adult relatives and levels of stigma are extremely high [12]. As study procedures posed minimal risk to participants, anonymised data from patients who were unable to consent were therefore also included. Consent was sought retrospectively from participants who regained capacity. All participants were free to withdraw at any time, and data were stored confidentially and anonymously.

All study protocols were approved by the Sierra Leone Ethics and Scientific Review Committee.

## Results

In total, 170 participants were screened (Table 1). 59% were female, with a median age of 36 years (IQR 30–43). At the time of testing, 54% were inpatients. 51% were newly diagnosed with HIV (defined as diagnosed with HIV less than 90 days prior to enrolment), and 56% were ART-naïve or had been newly initiated on ART (within the past 30 days), whereas 44% were ART-experienced. The median CD4 cell count was 45 cells/mm^3^ (IQR 23–63).

**Table 1:** Prevalence of cryptococcal antigenaemia (CrAg) by demographic and clinical characteristics

	Total number tested for CrAg (% of total)	Number CrAg positive	Prevalence of CrAg % (95%CI)	*P*-value (Fisher’s exact)
Total	170	8	4.7 (2.4–9.2)	
Sex				0.144
Male	69 (40.6)	1	1.4 (0.2–10.0)	
Female	101 (59.4)	7	6.9 (3.3–14.0)	
Age (years)				1.000
< 35	64 (37.6)	3	4.7 (1.5–13.9)	
≥35	106 (62.4)	5	4.7 (1.9–11.0)	
Admission status				0.287
Outpatient	79 (46.5)	2	2.5 (0.6–9.8)	
Inpatient	91 (53.5)	6	6.6 (2.9–14.1)	
CD4 (cells/mm^3^)				1.000
< 50	96 (56.5)	5	5.2 (0.7–9.7)	
≥ 50	74 (43.5)	3	4.1 (0.0–8.7)	
Time since HIV diagnosis (days)				0.278
< 90 “newly diagnosed”	86 (50.6)	6	7.0 (3.1–14.9)	
≥ 90	84 (49.4)	2	2.4 (0.6–9.5)	
Previous antiretroviral therapy (ART)^a^				0.468
ART naïve or newly-initiated	91 (56.2)	6	6.6 (2.9–14.1)	
ART experienced	71 (43.8)	2	2.8 (0.7–10.9)	
On ART at time of recruitment^a^				1.000
Yes	43 (26.9)	2	4.7 (1.1–17.6)	
No	117 (73.1)	6	5.1 (2.3–11.1)	

^a^ It was not possible to determine ART history for 8 participants (4.7%), or current ART status for 10 participants (5.9%); all of these were CrAg negative

Eight participants were CrAg positive, giving a prevalence of 4.7% (95% confidence interval 2.4–9.2). While this study was not powered to look at predictors of CrAg positivity, there were no significant predictors of antigenaemia, although there was a trend towards a higher prevalence amongst inpatients of 6.6% (95% CI 2.9–14.1) vs 1.8% in outpatients (95% CI 0.6–9.8) (*p* = 0.287).

Of the eight participants who were CrAg positive, seven (87.5%) were female, with a median age of 35 (IQR 27.5–37) (Table 2). Six (75%) were inpatients at the time of diagnosis, and six (75%) were newly HIV-diagnosed and ART-naïve. The remaining two participants were taking ART at the time of CD4 testing (viral load results unavailable). On review by a clinician, seven (87.5%) had a history of at least one of headache, confusion or decreased conscious level (75, 50, and 50% respectively). Four (50%) had features of meningitis on examination. All eight participants underwent a lumbar puncture. Four (50%) had a CSF opening pressure greater than 20cmCSF, and five (62.5%) had a positive CSF CrAg.

**Table 2:** Characteristics and outcomes of participants with positive blood cryptococcal antigen (CrAg)

Identifier	1	2	3	4	5	6	7	8
Age	36–45	26–35	26–35	18–25	26–35	18–25	36–45	46–55
Sex	Female	Female	Male	Female	Female	Female	Female	Female
Admission status at enrolment	Inpatient	Inpatient	Inpatient	Inpatient	Inpatient	Outpatient	Inpatient	Outpatient
CD4	23	56	74	12	55	28	0	15
Time since HIV diagnosis (days)	7	194	0	234	4	0	5	14
Antiretroviral therapy (ART) history	ART-naïve	Current ART (tenofovir + lamivudine+ efavirenz) duration 194 days	ART-naïve	Current ART (tenofovir + lamivudine + efavirenz) duration 97 days	ART-naïve	ART-naïve	ART-naïve	ART-naïve
Neurological symptoms at enrolment	Yes (headache, confusion, nausea)	Yes (headache, confusion, drowsy)	Yes (confusion, decreased conscious level)	No	Yes (headache, diplopia due to 6th nerve palsy)	Yes (mild headache)	Yes (headache, confusion, decreased conscious level, nausea)	Yes (mild headache)
Meningitic at enrolment	Yes	Yes	Yes	No	No	No	Yes	No
Glasgow Coma Score at enrolment	15	14	10	15	15	15	13	15
Lumbar puncture (LP) performed	Yes	Yes	Yes	Yes	Yes	Yes	Yes	Yes
Opening pressure	Raised	Normal	Raised	Normal	Raised	Normal	Raised	Normal
Cerebrospinal fluid CrAg	Positive	Positive	Negative (repeated; India Ink also negative)	Negative	Positive	Negative	Positive	Positive
Diagnosis	Cryptococcal meningitis	Cryptococcal meningitis	Cryptococcal antigenaemia; suspected TB meningitis	Cryptococcal antigenaemia	Cryptococcal meningitis	Cryptococcal antigenaemia	Cryptococcal meningitis	Sub-clinical cryptococcal meningitis
Number of additional therapeutic LPs performed	2	3	0	0	8	0	2	2
Outcome	Died	Died	Died	Died	Alive	Alive	Died	Alive
Time of outcome (days after enrolment)	3	5	1	0			20	
Clinical cause of death	Cryptococcal meningitis	Cryptococcal meningitis	TB, suspected TB meningitis	Sudden death, cause unknown. Sputum Xpert MTB-RIF positive.	NA	NA	Cryptococcal meningitis, sepsis	NA

The three participants with cryptococcal antigenaemia and a negative CSF CrAg were treated with oral fluconazole. CSF fungal cultures or polymerase chain reaction (PCR) were not performed as facilities for these techniques are not locally available. Two (67%) died as hospital inpatients at 0- and 1-days post-CrAg; both were confirmed as TB coinfected and neither participant was the cause of death felt clinically to be cryptococcal disease. The remaining participant was alive and established on ART at 8 weeks of follow-up. All five participants with cryptococcal meningitis were treated with high-dose fluconazole, and all received at least two therapeutic lumbar punctures. Three (60%) died at 3, 5 and 20 days after CrAg testing. Two (40%) were alive and established on ART at 8 weeks of follow-up.

## Discussion

Our study has demonstrated a prevalence of 4.7% (95% confidence interval 2.4–9.2) of cryptococcal infection among people with advanced HIV in a large tertiary hospital in Sierra Leone. To our knowledge, this is the first assessment of cryptococcal antigenaemia and cryptococcal disease-related mortality in this setting. This prevalence exceeds the previously recommended WHO threshold for the institution of routine screening for cryptococcal diseases [13]. In other published studies from West Africa, CrAg seroprevalence in PLHIV with CD4 < 100 cells/mm^3^ ranges from 2 to 21% [14–18]. However, heterogeneity between study populations (for example, whether they include only newly-diagnosed ART-naïve individuals, and/or only outpatients) and evidence of regional variation [17] prohibits direct comparison.

It was feasible to conduct CrAg screening in this setting. Currently, there is no national policy on diagnosis, management and prevention of cryptococcal disease in Sierra Leone, but 2018 WHO guideline on cryptococcal diseases strongly recommends CrAg screening for adults and adolescents with CD4 cell count < 100 cells/mm^3^ [2]. This intervention has been shown to be cost-effective and effective in reducing cryptococcal-related mortality [4, 19], and it is hoped that this evidence will contribute to wider adoption in Sierra Leone.

The overall mortality among CrAg positive participants within 8 weeks of follow up was 62.5%. Sadly, this is comparable to other studies from the region [14] and to the overall estimate of 70% one-year mortality for people in LMICs with cryptococcal meningitis [1]. One contributor to the high mortality in our study is likely to be the use of fluconazole monotherapy in the treatment of cryptococcal meningitis. At the time of study initiation, this was still listed as an alternative treatment by the WHO guidelines but is no longer recommended for the treatment of cryptococcal meningitis given the abundance of evidence for superior antifungal regimens [2, 20]. Despite the new WHO recommendation of dual antifungal therapy as an induction regimen for cryptoccocal meningitis [2], fluconazole remains the only available treatment for cryptococcal disease in Sierra Leone. We, therefore, add our voices to those advocating for effective treatments for cryptococcal disease to be made more accessible to PLHIV in low-income countries [21]. Routinely screening patients with advanced HIV disease for CrAg as part of their pre-ART interventions and the provision of appropriate dual antifungal regimen should be instituted in Sierra Leone and similar countries to prevent the unwarranted morbidity and mortality [2, 22].

Given that several of the deceased participants were felt clinically to have a cause of death other than cryptococcal disease, it is important to deliver a “package” of care to those with advanced HIV (CD4 cell count < 200 cells/mm^3^) [23] which includes CrAg screening and other aspects including tuberculosis screening and prevention, and rapid ART initiation. Sierra Leone has recently operationalised a differentiated HIV service delivery guide which includes provisions directed towards people with advanced HIV.

Additional measures are also needed to reduce cryptococcal mortality in Sierra Leone. In a recent study, 21% of people with newly-diagnosed HIV in Freetown had a CD4 cell count below 100 cells/mm^3^, suggesting a need for earlier HIV testing, particularly given the limited resources for managing opportunistic infections [24]. Similar to other studies in sub-Saharan Africa [25], a significant proportion of participants in this study were ART-experienced, either with presumed treatment failure, poor adherence, or loss from care. This calls for prudent programmatic activities to improve treatment adherence. Many studies of cryptococcosis prevention exclusively focus on ART-naïve populations, but our finding that two (25%) of those with cryptococcal antigenaemia were ART-experienced suggests that CrAg screening should not be restricted to newly diagnosed HIV. PLHIV with virologic or immunologic treatment failure and those with symptoms who report after treatment interruptions should be considered for routine CrAg screening if their CD4 cell count is < 200 cells/mm^3^.

Our study has several limitations. First, as a single-site study in a tertiary hospital, it may not be representative of Sierra Leone with respect to both patient characteristics and feasibility of implementing screening. Secondly, 54% of participants were inpatients and those who presented with neurological symptoms were not excluded. Therefore, they may not truly represent the impact of a screening programme (ideally targeting asymptomatic outpatients.) However, this study was conducted in the context of implementation of a reflexive CrAg testing programme, and the study population does reflect those tested for CD4 cell count at the centre. Lack of fungal cultures and other diagnostic tests did not make it possible to confirm diagnoses of cryptococcosis or meningitis; nor was it possible to definitively establish the cause of death in deceased CrAg-positive participants.

## Conclusion

Our study demonstrated a significant burden of cryptococcal disease at Connaught Hospital in Sierra Leone. We recommend for the national HIV programme and its partners to formulate and implement policies on screening and pre-emptive fluconazole therapy for cryptococcal diseases as part of a package of differentiated care for people with advanced HIV, in conjunction with programmatic strategies to improve HIV diagnosis and virological suppression. Affordable access to adequate antifungal regimens is required to improve outcomes for cryptococcal disease in sub-Saharan Africa.

Finally, antiretroviral treatment interruptions among the cohort of patients in this study are high. In order to prevent this, appropriate psychosocial counselling techniques and other programmatic strategies are needed.

## Data Availability

The data supporting this article is available in the repository of University of Sierra Leone and will be made easily available on request to the corresponding author when required.

## Abbreviations

ART: Antiretroviral therapy
ARVs: Antiretrovirals
CD4: Cluster determinant 4
CrAg: Cryptococcal Antigen
CSF: Cerebrospinal fluid
HIV: Human Immunodeficiency Virus
LMIC: Low- and Middle-Income Countries
PLHIV: People Living with HIV
SLESRC: Sierra Leone Ethics and Scientific Review Committee
WHO: World Health Organization

## References

[1] Rajasingham R, Smith RM, Park BJ, Jarvis JN, Govender NP, Chiller TM (2017). Global burden of disease of HIV-associated cryptococcal meningitis: an updated analysis. Lancet Infect Dis.

[2] WHO | Guidelines for the diagnosis, prevention and management of cryptococcal disease in HIV-infected adults, adolescents and children. WHO. 2018; Available from: apps.who.int/iris/bitstream/handle/10665/260399/9789241550277-eng.pdf;jsessionid=30132897E0F565C43DF9DDFD1B0AFA3F?sequence=1. Accessed 13 Dec 2019. 30285342

[3] Meya DB, Manabe YC, Castelnuovo B, Cook BA, Ali M, Kambugu A (2010). Serum Cryptococcal antigen (CRAG) screening is a cost- effective method to prevent death in HIV- infected persons with CD4 <100/uL starting HIV therapy in resource-limited settings. Clin Infect Dis.

[4] Jarvis JN, Harrison TS, Lawn SD, Meintjes G, Wood R, Cleary S (2013). Cost effectiveness of Cryptococcal antigen screening as a strategy to prevent HIV-associated Cryptococcal meningitis in South Africa. PLoS One.

[5] Hakim J, Musiime V, Szubert AJ, Mallewa J, Siika A, Agutu C (2017). Enhanced prophylaxis plus antiretroviral therapy for advanced HIV infection in Africa. N Engl J Med.

[6] Parkes-Ratanshi R, Wakeham K, Levin J, Namusoke D, Whitworth J, Coutinho A (2011). Primary prophylaxis of cryptococcal disease with fluconazole in HIV-positive Ugandan adults: a double-blind, randomised, placebo-controlled trial. Lancet Infect Dis.

[7] Sierra Leone Demographic and Health Survey. 2013; Available from: www.statistics.sl/images/StatisticsSL/Documents/demographic_and_health_survey_2013_final-report.pdf. Accessed 10 Feb 2020.

[8] WHO | WHO Global Health Workforce Statistics. WHO. 2018; Available from: www.who.int/hrh/statistics/en/. Accessed 12 Dec 2019.

[9] Gaskell Katherine M., Rothe Camilla, Gnanadurai Roshina, Goodson Patrick, Jassi Chikondi, Heyderman Robert S., Allain Theresa J., Harrison Thomas S., Lalloo David G., Sloan Derek J., Feasey Nicholas A. (2014). A Prospective Study of Mortality from Cryptococcal Meningitis following Treatment Induction with 1200mg Oral Fluconazole in Blantyre, Malawi. PLoS ONE.

[10] Boyles Tom H., Gatley Elizabeth, Wasserman Sean, Meintjes Graeme (2017). Brief Report. JAIDS Journal of Acquired Immune Deficiency Syndromes.

[11] Molyneux E (2013). Where there is no laboratory, a urine patch test helps diagnose meningitis. J Neurosci Rural Pract.

[12] World Health Organization. Sierra Leone Sierra Leone. Time. 2016, 2003;(January):–4.

[13] Organization WH. Rapid advice: diagnosis, prevention and management of cryptococcal disease in HIV-infected adults, adolescents and children: December 2011. Geneva World Heal Organ. 2011;(December) Available from: http://apps.who.int/iris/handle/10665/44786. 26110194

[14] Thomsen D, Hviid CJ, Hønge BL, Medina C, da Silva Té D, Correira FG, et al. Increased mortality among HIV infected patients with cryptococcal antigenemia in Guinea-Bissau. Pan Afr Med J. 2018. 10.11604/pamj.2018.29.18.14099 PMC5899773 29662603

[15] Osazuwa F, Dirisu JO, Okuonghae PE, Ugbebor O (2012). Screening for cryptococcal antigenemia in anti-retroviral naïve AIDS patients in Benin City, Nigeria. Oman Med J.

[16] Oladele RO, Akanmu AS, Nwosu AO, Ogunsola FT, Richardson MD, Denning DW (2016). Cryptococcal antigenemia in Nigerian patients with advanced human immunodeficiency virus: Influence of antiretroviral therapy adherence. Open Forum Infect Dis.

[17] Ezeanolue EE, Nwizu C, Greene GS, Amusu O, Chukwuka C, Ndembi N (2016). Brief report: geographical variation in prevalence of Cryptococcal Antigenemia among HIV-infected, treatment-naive patients in Nigeria: a multicenter cross-sectional study. J Acquir Immune Defic Syndr.

[18] Mamoojee Y, Shakoor S, Gorton RL, Sarfo S, Appiah LT, Norman B (2011). Short communication: low seroprevalence of cryptococcal antigenaemia in patients with advanced HIV infection enrolling in an antiretroviral programme in Ghana. Tropical Med Int Health.

[19] Meya D, Rajasingham R, Nalintya E, Tenforde M, Jarvis JN (2015). Preventing Cryptococcosis—shifting the paradigm in the era of highly active antiretroviral therapy. Curr Trop Med Reports.

[20] WHO. Rapid Advice: Diagnosis, Prevention and Management of Cryptococcal Disease in HIV-Infected Adults, Adolescents and Children. 2011. Available from: apps.who.int/iris/bitstream/handle/10665/44786/9789241502979_eng.pdf?sequence=1&isAllowed=y. Accessed 09 Feb 2020. 26110194

[21] Loyse A, Burry J, Cohn J, Ford N, Chiller T, Ribeiro I, et al. Leave no one behind: response to new evidence and guidelines for the management of cryptococcal meningitis in low-income and middle-income countries. Lancet Infect Dis. 2018; Available from: www.dndi.org/wp-content/uploads/2018/10/Loyse_Response_Evidence_Guidelines_CryptococcalMeningitis_LancetID_2018.pdf. 10.1016/S1473-3099(18)30493-6 30344084

[22] Vaidhya SA (2015). Combination versus Monotherapy for the treatment of HIV associated Cryptococcal meningitis. J Clin Diagnostic Res [Internet].

[23] WHO (2017). HIV Treatment. Guidelines for managing advanced HIV disease and rapid initiation of ARV.

[24] Yendewa GA, Poveda E, Lakoh S, Yendewa SA, Jiba DF, Salgado-Barreira A (2018). High prevalence of late-stage disease in newly diagnosed human immunodeficiency virus patients in Sierra Leone. Open Forum Infect Dis.

[25] Ousley J, Niyibizi AA, Wanjala S, Vandenbulcke A, Kirubi B, Omwoyo W (2018). High proportions of patients with advanced HIV are antiretroviral therapy experienced: hospitalization outcomes from 2 sub-Saharan African sites. Clin Infect Dis.

